# On the Reliability of the Notion of Native Signer and Its Risks

**DOI:** 10.3389/fpsyg.2022.716554

**Published:** 2022-03-14

**Authors:** Giorgia Zorzi, Beatrice Giustolisi, Valentina Aristodemo, Carlo Cecchetto, Charlotte Hauser, Josep Quer, Jordina Sánchez Amat, Caterina Donati

**Affiliations:** ^1^Department of Translation and Language Sciences, Universitat Pompeu Fabra, Barcelona, Spain; ^2^Department of Psychology, University of Milano-Bicocca, Milan, Italy; ^3^Université Paris Cité, CNRS, Laboratoire Linguistique Formelle UMR 7110, Paris, France; ^4^UMR 7023 Structures Formelles du Langage, Paris, France; ^5^ICREA – Pompeu Fabra University, Barcelona, Spain

**Keywords:** sign languages, native signer, early and late signers, effect of age of exposure, language assessment

## Abstract

Who is a native signer? Since around 95% of deaf infants are born into a hearing family, deaf signers are exposed to a sign language at various moments of their life, and not only from birth. Moreover, the linguistic input they are exposed to is not always a fully fledged natural sign language. In this situation, is the notion of native signer as someone exposed to language from birth of any use? We review the results of the first large-scale cross-linguistic investigation on the effects of age of exposure to sign language. This research involved about 45 Deaf adult signers in each of three sign languages (Catalan Sign Language, French Sign Language, and Italian Sign Language). Across the three languages, participants were divided into three groups – those exposed from birth, those between 1 and 5 years of age, and those exposed between 6 and 15 years of age – and received a battery of tests designed for each language targeting various aspects of morphosyntactic competence. In particular, the tests focused on both those morphosyntactic phenomena that are known from the spoken language literature to be good detectors of language impairment or delay (i.e., *wh-*interrogatives and relative clauses) and on morphosyntactic phenomena that are sign language specific (i.e., role shift and directional verbs). The results showed a clear effect of being native, with significant differences across languages and tests between signers exposed to sign language from birth and those exposed in the 1st years of life. This confirms the life-long importance of language exposure from birth and the reliability of the notion of “*nativeness*”, at least for syntax. On the other hand, while in most domains the differences observed between populations might be differences in performance, for some specific constructions, signers belonging to the three groups may have different grammars. This latter finding challenges the generalized use of native signers’ grammar as the baseline for language description and language assessment.

## Introduction

The notion of “native” user of a language has become controversial for various reasons. For spoken languages, the challenge comes from bilingualism and multilingualism (Sorace, 2021 for an overview), while for sign languages the controversy is due to the unique sociolinguistic situation that characterizes the population of Deaf signers.^1^ The linguistic profiles attested among deaf people are very diverse, and native signers, defined as deaf individuals who were born into a Deaf signing family, are only a small minority. This led many scholars to challenge the importance of this notion as a reliable criterion for language description and assessment, at least as far as sign languages are concerned. The question is whether “*nativeneness*” is indeed different from early exposure: in other words, whether what really matters is being early exposed to a sign language, or whether there is a special status associated to being native, even with respect to early learners.

In this paper we will first discuss the controversial status of “native signers” with respect to the global population of Deaf signers, underlying that most experimental studies have not been using consistent criteria to contrast native signers from those signers who were not exposed to a sign language from birth. With the goal of contributing with experimental methods to the debate of whether native and non-native signers are indeed different, in the section “Native, Early and Late Signers in a Large-Scale Cross-Linguistic Investigation” we will present an overview of the morpho-syntactic comprehension tests developed within the Horizon 2020 project SIGN-HUB (“The Sign Hub: preserving, researching and fostering the linguistic, historical and cultural heritage of European Deaf signing communities with an integral resource”) describing the criteria that were used to select the groups of native, early and late signers, and the tests themselves, and providing a summary of the results. In the section “Discussion”, we will then discuss the results presented in the previous section and support the claim that native signers have indeed a different performance in comparison to signers who were exposed later to sign language, even early in life. At the same time, we will challenge the reliability of native signers’ grammar as the baseline to be used for sign language investigation and assessment.

## The Controversial Notion of “Native Signer”

The population of deaf pre-lingual adult signers is extremely heterogeneous, as it is characterized by individuals with very different linguistic backgrounds (Johnston, 2006; Costello et al., 2008; Quer and Steinbach, 2019). This is due to the sociolinguistic situation that characterizes deaf people and Deaf communities. The general estimation is that only 5–10% of deaf babies have deaf signing parents, and even less have deaf signing grandparents (Newport, 1988; Neidle et al., 2000; Mitchell and Karchmer, 2004) and therefore only a small part of the deaf population is exposed to sign language from birth. This percentage, however small, has been calculated on the deaf population of the United States, but it has been questioned as an overestimation for the deaf population in Australia (Johnston, 2006) and Europe (Costello et al., 2008). Costello et al. (2008) looked at deaf signers in the Basque Country, in the north of Spain, underlining that the number of deaf people born into Deaf families is extremely low and it hardly reaches 5%.^2^ This aspect needs to be taken into consideration especially when looking at deaf populations in smaller communities.

### How Deaf Children Get Exposed to Language

If we consider the general definition of native signers as “Deaf people who grew up with Deaf signing parents and who identify with the Deaf community” (Neidle et al., 2000), it is clear that it refers to a very small part of the deaf population. In addition to this, it is important to remark that some deaf parents might have been themselves exposed to sign language at a late point in life and therefore might provide a language input to the child that cannot be strictly compared to the one of a native (Lillo-Martin, 2021). Even if it has been shown that deaf children exposed to a non-native sign language from birth reach a better performance than their parents and get close to their native peers, they are still not native-like (Singleton and Newport, 2004). Deaf children exposed to a native input might thus be exceedingly rare.

As for the rest of the deaf children population, it is constituted of deaf children born in hearing families, and therefore for the most part they are not exposed to sign language from birth. There are several factors that prevent the deaf population from being exposed to an early and adequate sign language input that would allow an early and natural language acquisition. The main reasons are: the age of diagnosis, although it has recently drastically decreased due to newborn hearing screening (Joint Committee on Infant Hearing, 2019), the different degrees of deafness, and the use of technologies such as cochlear implants or hearing aids, together with the type of language intervention adopted by the parents: they might opt for exposure to spoken language alone *via* amplification through hearing aids or cochlear implants, or rather for exposure to both spoken and sign languages or for exposure to sign language only. In many cases parents are advised by doctors and educators to adopt an oralist approach supporting the use of technologies meant to facilitate the learning of spoken languages, denying sign input (Lillo-Martin, 2021). Even with early intervention through hearing aids or cochlear implants, though, language access is delayed if not provided through a fully accessible input, which in the case of deaf children is in the visual-gestural modality (Humphries et al., 2016; Hall et al., 2019, among others). In a very small percentage of cases, hearing parents decide to learn sign language and expose their child to it (cf. Chen Pichler and Lillo-Martin, 2018), hence still delaying giving a sign language input while they go through the process of learning the language. Eventually, the input they provide cannot be compared to the one of deaf native signers (Lillo-Martin, 2021), even though it is provided early in life. Only a very small minority of deaf children born in hearing families is then exposed to sign language in their parental home shortly after diagnosis. In most cases, it is only in school that deaf children get exposed to sign language.

A delayed exposure to sign language leads to a delay in the development of language, and even to atypical neurological mappings of language (Mayberry, 2010; Mayberry and Kluender, 2018; Woll, 2018). Moreover, it has sociolinguistic consequences in relation to how deaf people not exposed to sign language from birth relate to the Deaf community. For German Sign Language (DGS), Jaeger (2019) distinguishes between a “native” and an “authentic” signer. Many participants to her study who were non-native reported that they identify themselves as “authentic signers”, specifying that such status can be reached either by being born into the Deaf community (“Deaf aristocracy”), or *via* intentional change (“Deaf meritocracy”), as was the case for most of them. This perspective on non-native signers as being native-like from an identity perspective relates to the conceptualization of non-native signers as “New Signers”, a concept adapted from that of “New Speaker”. The term “New Speaker” was introduced to indicate users who acquire a minority language later in life and outside the parental home (O’Rourke et al., 2015), especially in the context of language revitalization (Jaffe, 2015). It has been recently extended to deaf non-native signers since they share the characteristics of acquiring language after childhood and outside the parental home (Jaeger, 2019) and because of the status of sign languages as minority languages (Bauman and Murray, 2017; Tupi, 2019). The New Signer model gives a new perspective to the “native speaker” ideology and shows that it is important to disentangle sociological and psycholinguistic factors when it comes to identifying the profile of a native signer.

Studying a sign language by only relying on native signers might end up as an impossible task. The alternative that has been adopted in the literature is to work with consultants that fulfill several criteria that make them as close as possible to the standard definition of native signers (Quer and Steinbach, 2019). As reported by Costello et al. (2008), many research groups tend to select participants, especially for neurolinguistic studies, who are (at least) second generation deaf-of-deaf signers. On the other hand, Mathur and Rathmann (2006) consider three main criteria: (i) exposure to sign language by the age of three; (ii) ability to give grammaticality judgments with ease; and (iii) daily contact with a sign language in the Deaf community for more than 10 years. In experimental data assessing language acquisition and the impact of age of exposure (AoE) on language competence, native signers tend to be strictly identified with individuals who have been exposed to sign language from birth from Deaf signing parents. Oftentimes, though, a limit of 3 years of age is established to consider someone as native (Mayberry, 1993; Freel et al., 2011).

It is clear that determining the exact criteria that define an individual as having native competence is particularly crucial when the aim is to assess the consequences that a delay to language exposure can cause earlier or later in life. In the following section, we provide an overview of the profiles of deaf signers that have been studied in this type of studies. In many cases, their goal is to determine whether native signers, even if they constitute a minority, can be distinguished from signers who have been exposed to sign language even quite early in life, as far as language development is concerned.

### Age of Exposure to Sign Language

Early exposure to language is crucial for language acquisition (Mayberry et al., 2002) and this has been documented for sign languages since the ’90s, with studies showing that non-native signers differ from native signers in several morpho-syntactic tasks. Emmorey et al. (1995), in a study on sign recognition within a sentence containing errors in verb agreement, showed that only native signers were sensitive to agreement errors, while late learners were not. The relevant group of late learners were exposed to American Sign Language (ASL) between 4 and 20 years of age. In a second experiment involving sign recognition in a sentence containing errors in verb agreement or aspect and offline grammaticality judgments, non-native signers were distinguished into early and late learners, with AoE range of 2–7 years and 10–20 years, respectively. The results of the first experiment were confirmed, with native signers outperforming non-native regardless of their AoE group. In other studies on ASL, though, the AoE effect was gradient, showing a continuum across the groups: as AoE increased, the performance of signers decreased. This is the case of a study on ASL sentence processing measured by recall of long and complex sentences. In this study, Mayberry (1993) included three groups of pre-lingual deaf signers with AoE ranging from (i) 0–3 years of age, (ii) 5–8 years, and (iii) 9–13 years (and a fourth group of post-lingual deaf signers who were exposed to ASL between 8 and 15 years of age and lost their hearing between 8 and 12 years of age). The performance of the pre-lingual deaf signers decreased as AoE increased. A similar result was obtained using a grammaticality judgment task on sentences of various types, independently from the syntactic structure investigated (Boudreault and Mayberry, 2006). In the same task, reproduced by Cormier et al. (2012) in British Sign Language (BSL), accuracy in the grammaticality judgment task decreased as AoE increased for Deaf early signers, while no decreasing related to AoE was found among Deaf late signers. However, if we compare the AoE of late learners in the two versions of the study, we observe that while in the ASL experiment late learners were exposed to ASL between 8 and 13 years, in the BSL experiment late learners were exposed to BSL between 9 and 18 years. More importantly, late ASL signers were described as L1 signers, whereas Cormier et al. (2012) suggest that their group of late signers was composed of L2 signers, with English as L1. The upward trend for the oldest AoE was then attributed to having acquired another language from birth.

The characteristics of the various groups of signers participating in the experiments just presented are summarized in Table 1.

**TABLE 1:** Summary of the age of exposure (AoE) of participants across a selection of relevant studies on the impact of AoE.

Language	Task	Participants
ASL	Sign recognition in a sentence containing errors in verb agreement (Emmorey et al., 1995)	(i) 11 Native: AoE = birth, Age = 21–44
		(ii) 10 Late: AoE = 4–20 (*M* = 12), Age = 29–49
	Sign recognition in a sentence containing errors in verb agreement or aspect and offline grammaticality judgments (Emmorey et al., 1995)	(i) 10 Native: AoE = birth, Age = 19–24
		(ii) 10 Early: AoE = 2–7 (*M* = 4), Age = 21–37
		(iii) 10 Late: AoE = 10–20 (*M* = 14), Age = 22–46
	Sentence processing (Mayberry, 1993)	(i) 9 AoE = 0–3 (*M* = birth), M Age = 51 (43–67), M SLe = 51 (43–67), born deaf
		(ii) 9 AoE = 5–8 (*M* = 7), M Age = 61 (37–71), M SLe = 51 (31–65), born deaf
		(iii) 9 AoE = 9–13 (*M* = 11), M Age = 60 (40–72), M SLe = 54 (28–61), born deaf
		(iv) 9 AoE = 8–15 (*M* = 11), M Age = 60 (38–72), M SLe = 50 (29–61), onset deafness: 8–12 (*M* = 9)
	Grammaticality judgment task on sentences (Boudreault and Mayberry, 2006)	(i) 10 Native: AoE = birth, M age = 24.2 (18–41), M SLe = 24.3 (18–41)
		(ii) 10 Early: AoE = 5–7 (*M* = 5.6), M Age = 43.2 (31–62), M SLe = 37.6 (14–47)
		(iii) 10 Late: AoE = 8–13 (*M* = 10.3), M Age = 43 (24–79), M SLe = 32.9 (13–71)
BSL	BSL version of Boudreault and Mayberry’s task (Cormier et al., 2012)	(i) 10 Native: AoE = birth, M Age = 39.7 (20–57), M SLe = 39.7 (20–57)
		(ii) 11 Early: AoE = 2–8 (*M* = 4.4), M Age = 36.5 (19–54), M SLe = 32 (17–51)
		(iii) 9 Late: AoE = 9–18 (*M* = 12.8), M Age = 30.9 (20–43), M SLe = 18.1 (10–26)

From Table 1, focusing on AoE, we can clearly see that there is a lot of variation across studies on the groups of signers investigated and how they are defined: in some cases, native signers are compared directly to late learners. In other cases, when three populations are indeed distinguished, the AoE range of the three groups varies a lot. It is possible to see variation in the definition of early and late signers even in the “replication” of the same study (cf. Boudreault and Mayberry, 2006, and Cormier et al., 2012). Moreover, in Mayberry (1993) the category with the earliest exposure to ASL includes signers who were exposed before 3 years of age, without excluding native signers from this sample.

Under these circumstances, it is thus not straightforward to compare the results of the various studies. In particular, it is not clear whether the effect of AoE found in the literature so far is a simple effect of being early exposed to a sign language, or whether there is a special status associated to being exposed from birth even with respect to early learners.

## Native, Early and Late Signers in a Large-Scale Cross-Linguistic Investigation

A large-scale cross-linguistic investigation was conducted within the SIGN-HUB project. With the aim of investigating the role of AoE in language comprehension in adulthood, four morpho-syntactic comprehension tests were developed in three different sign languages (Catalan Sign Language (LSC), French Sign Language (LSF), and Italian Sign Language (LIS)]. Results of those tests, separately discussed in Aristodemo et al. (2020, in press), Cecchetto et al. (2021), Hauser et al. (2021, in press), are crucial to understanding whether native and non-native signers differ categorically, or whether what matters is simply early exposure to sign language for which we expect a gradient effect associated to different AoE groups.

### The Participants in the SIGN-HUB Tests

In the SIGN-HUB tests, for the three languages (LIS, LSC, and LSF), participants were selected following three general inclusion criteria: (i) onset of deafness not later than 3 years of age;^3^ (ii) first exposure to sign language not later than 15 years of age; and (iii) the target sign language as their preferred mean of communication. All participants had been exposed to sign language for at least 15 years, with the exception of two young LSF participants, who both had only 9 years of sign language experience.

To be able to create groups of participants with a similar language input and background, they were asked to fill in a questionnaire containing several personal questions including AoE, the possible deafness of their parents, whether their parents were signers, whether they went to a school for the deaf or had deaf school mates, and so on.^4^ Participants were divided into three groups: (i) native, (ii) early, and (iii) late signers. Native signers were individuals exposed to sign language from birth (AoE = 0), having at least one deaf signing parent, and who therefore acquired SL in a family environment. Early learners were exposed to sign language between 1 and 5 years of age while late learners between 6 and 15 years of age. The choice of the age ranges was based (i) on including among native signers only those people exposed to a sign language from birth; (ii) on having in the early learners group signers who were exposed to a sign language very early in life or at least within the critical acquisition period up to 5 years of age, but not from birth; and (iii) on comprising in the late learners group signers who were exposed to sign language not later than 15 year old, which is the average age limit for being exposed to a sign language in a school setting in the target language countries. In both groups of non-native signers, most participants were introduced to sign language in institutional educational settings (preschool for early signers and school for late signers), almost none had deaf parents, and very few had at least one parent knowing sign language. Table 2 summarizes the characteristics of all participants that were considered for the first participant selection.

**TABLE 2:** Summary of SIGN-HUB participants’ characteristics per group and language.

Group	SL	N.	AoE	Everyday use of SL	Deaf parent(s)	Signing parent(s)	Context of exposure to SL	Years of SL experience
NATIVE	LIS	16	0	16	16	16	Family: 16	30–60 (*M* = 43)
	LSC	14	0	13^a^	14	14	Family: 14	26–69 (*M* = 44)
	LSF	14	0	13^b^	13	13	Family: 13 (1 NS)	26–54 (*M* = 39)
EARLY	LIS	15	2–5 yrs (*M* = 3,9)	13	1	3	Family: 4 Preschool: 10 (1 NS)	32–58 (*M* = 47)
	LSC	16	3–5 yrs (*M* = 3.5)	15	1	2	Family: 3 Preschool: 13	20–60 (*M* = 48)
	LSF	15	1–5.5 yrs (*M* = 3.4)	10	none	1	Family: 3 Preschool: 11 (1 NS)	20–39 (*M* = 30)
LATE	LIS	13	6–15 yrs (*M* = 9.1)	11	none	1	Family: 2 School: 9 (2 NS)	26–58 (*M* = 41)
	LSC	12	6–15 yrs (*M* = 10.4)	11	1	2	School: 8 (4 NS)	34–57 (*M* = 41)
	LSF	14	6–14 yrs (*M* = 9.2)	11	2	1	Family: 1 School: 9 (4 NS)	9–63 (*M* = 31)

*^a^For LSC, one native signer, one early and one late declared to use LSC “often” instead of “everyday”.*

*^b^For LSF, one native, five early and three late signers declared to use LSF “often” instead of “everyday”.*

The questionnaires participants filled in were also used to collect more general personal information. Table 3 summarizes data of the final pool of participants considered in the analyses about chronological age (inserted as a factor in the various analyses), gender, degree of deafness, and use of hearing aids. The questionnaire was meant also to collect information about participants’ use of written language (either Italian, Catalan, Spanish, or French). They were asked to self-rate whether they used written language every day, and if they read newspapers, etc. However, the data obtained, which might be considered as an indirect measure of their competence in the spoken language, were often not coherent and in any case not fine-grained enough to be used as a factor in the analyses.

**TABLE 3:** Summary of SIGN-HUB participants’ general characteristics per group and language.

Group	SL	N.	Age	Gender	Degree of deafness^9^	Hearing aids	Education
NATIVE	LIS	16	30–60 (*M* = 43)	10 female 6 male	15 very severe 1 moderate	6 hearing aids	Median = high school
	LSC	14	26–69 (*M* = 44)	7 female 7 male	13 very severe 1 moderate	None	Median = university education
	LSF	14	26–54 (*M* = 39)	6 female 8 male	9 very severe 5 severe	7 hearing aids 1 cochlear implant	Median = middle school
EARLY	LIS	15	34–62 (*M* = 48)	7 female 9 male	14 very severe 1 severe	5 hearing aids	Median = high school
	LSC	16	23–64 (*M* = 51)	10 female 6 male	16 very severe	None	Median = middle school
	LSF	15	24–47 (*M* = 34)	10 female 5 male	13 very severe 2 severe	4 hearing aids 1 cochlear implant	Median = university education
LATE	LIS	13	40–65 (*M* = 50)	4 female 9 male	10 very severe 2 severe 1 moderate	3 hearing aids 1 cochlear implant	Median = high school
	LSC	12	41–63 (*M* = 52)	5 female 7 male	10 very severe 2 severe	5 hearing aids	Median = middle school
	LSF	14	19–72 (*M* = 40)	8 female 6 male	12 very severe 2 severe	6 hearing aids 1 cochlear implant	Median = high school

*^9^Following the recommendation by the International Bureau for Audiophonology BIAP, “very severe” is considered a degree of deafness higher than 90 dB, “severe” a degree between 71 and 90 dB, and “moderate” a degree of deafness between 41 and 70 dB.*

**FIGURE 1:**
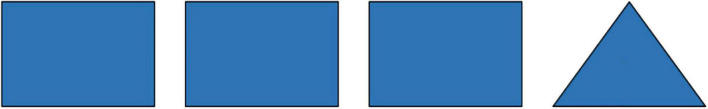
Example of one item of the Odd One Out Cognitive Task (Hauser et al., 2021: 18). (CC-BY 4.0).

All participants were screened for cognitive deficits, using the Odd One Out Cognitive Task (Giustolisi and Friedmann, 2019, for LIS, Zorzi et al., 2019c, for LSC and Aristodemo and Friedmann, 2019, for LSF), which was designed to detect potential cases of cognitive impairment. In this test, participants needed to find the intruder in a set of four pictures (see Figure 1 for an example). The Odd One Out Cognitive Task displayed 28 items preceded by two training items. For each participant, *z*-scores were calculated considering language group mean and standard deviations. Participants with *z*-scores lower than −2.5 were excluded from the study.

One native participant was excluded both from the LIS pool and from the LSC pool. The LIS participant had a z-score of −3.94 and the LSC one a z-score of −5.51. No participant was excluded from the LSF pool.

### The SIGN-HUB Tests

The SIGN-HUB project tests had two main goals: (i) providing data for the understanding of the effect of AoE in signers, and (ii) contribute to the comparative analysis of some specific linguistic phenomena. They were developed to study complex structures of two types: either characterized by long-distance dependencies and known to be good detectors of language impairment or delay (i.e., relative clauses and *wh-*questions), as in Friedmann et al. (2009), or prototypical sign language modality specific constructions (i.e., role shift and expression of agreement through directional verbs).^5^ Each test was language specific, but they were similar in design and, most importantly, the criteria to distinguish the populations investigated were the same.^6^

#### Long Distance Dependencies: Relative Clauses and *Wh*-Questions

For head initial languages such as English it has been found that subject relative clauses are easier to understand than object relative clauses, and this is also the case for subject *wh-*interrogatives with respect to object *wh-*clauses (Friedmann et al., 2009, among others). Such asymmetry, that goes under the name of Subject Advantage, has been accounted in various ways, with proposals pointing at resource-based effects related to structural distance (Frazier, 1987; Hawkins, 1999), intervention (Friedmann et al., 2009), linear distance (e.g., King and Just, 1991; Gibson, 2000), canonical order effects (Diessel and Tomasello, 2005), distribution-based effects (e.g., Mak et al., 2006), and prominence-factors (Van Valin and Wilkins, 1996). Most studies point toward a universal Subject Advantage at the cross-linguistic level, but interestingly, most of them focus on head initial languages. In the SIGN-HUB project tests, LSF allows both SOV and SVO orders with preference varying across individuals (Hauser, 2019), while LIS and LSC show an SOV order (Quer, 2002; Cecchetto et al., 2006). Moreover, among the three languages, different strategies are used to realize relative clauses and *wh-*constructions: LSF has head-external relative clauses and *in situ wh-*interrogatives (Hauser, 2019), while LIS and LSC have head-internal relative clauses and *wh-*clauses involving *wh-*movement to the right periphery of the clause (Quer et al., 2005; Branchini and Donati, 2009; Cecchetto et al., 2009; Mosella Sanz, 2012). In addition to providing new results contributing to the debate of age of language exposure as a factor in language assessment, which we shall discuss here, the SIGN-HUB tests also provide crucial data from a different modality on how to explain the Subject Advantage from a theoretical point of view. We refer to Cecchetto et al. (2021), Hauser et al. (2021, in press) for a detailed discussion of these conclusions.

**FIGURE 2:**
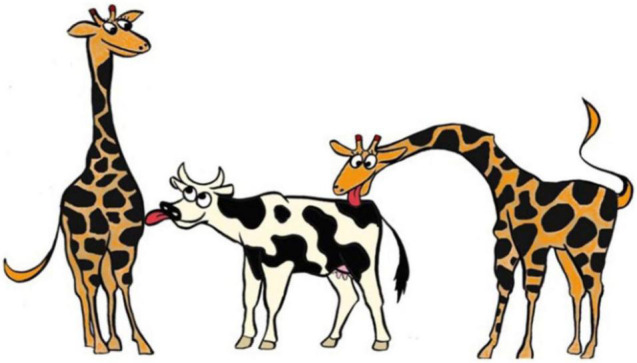
Example of a three characters picture (Hauser et al., 2021: 14). (CC-BY 4.0).

Concerning the SIGN-HUB tests on relative clauses (Hauser et al., 2021), they aimed at investigating the comprehension of subject and object relative clauses in a sentence-to-picture matching task based on Friedmann et al. (2009). In each picture, three characters were displayed: two identical characters either performing an action or undergoing that action with respect to a third different character standing between them (Figure 2).^7^

The same picture was used to match a subject RC (i.e., “Choose the lion that licks the dog”) or an object RC (i.e., “Choose the lion that the dog licks”). Participants were asked to choose one of the characters depending on the type of relative clause they were watching.

As for *wh-*interrogatives (also called content questions), the SIGN-HUB tests aimed at assessing comprehension of subject and object questions in a similar sentence-to-picture matching task (Cecchetto et al., 2021; Hauser et al., in press). The pictures also displayed three characters, like the one in Figure 2, and the answer to the question always targeted the characters on the sides in order to test the subject and object conditions (i.e., “Who licks the cow?” and “Who does the cow lick?,” respectively).

#### Modality Specific Phenomena: Role Shift and Agreement

Two comprehension tests within the SIGN-HUB project were created to investigate two constructions that are modality specific: role shift and the expression of spatial agreement through directional verbs. Role shift is commonly used in sign languages and is particularly interesting for its semantic properties; spatial agreement consists in a strategy expressing agreement through articulation in space of the trajectory associated with the verb. This latter phenomenon has been studied in other sign languages showing an important impact of AoE (Emmorey et al., 1995; Cormier et al., 2012, among others). The two tests were language specific but had a similar design across languages (Aristodemo et al., 2020, in press).

Role shift (RS) is a construction commonly used in sign languages to report utterances or thoughts from the perspective of an agent distinct from the utterance speaker (Quer, 2011). It is signaled by specific non-manual markers that can slightly vary across languages, but that in general are characterized by body/head movement toward the locus in space assigned to the referent whose utterance or thought has been reported, and eye-gaze contact break with the actual addressee. Interestingly, when introduced by a verb like SAY, but also when no introducing predicate is used, role shift displays indexical shift: indexical expressions like the first-person pronoun (IX_1_) retrieve their reference from the reported context. One of the main goals of these tests was to assess the comprehension of pairs of sentences with and without role shift with a first-person pronoun embedded under SAY. Like the other tests presented so far, this study on role shift was also meant to make a contribution to the debate on the theoretical nature of this structure. The tests were a sentence-to-picture matching task, in which participants were asked to pick one of two pictures matching a target sentence. Crucially, the choice depended on whether participants shifted (or not) the referent of the first-person pronoun in the target sentence.

As for the assessment of comprehension of agreement using directional verbs, this type of verbs are characterized by the articulation of a trajectory in the signing space from the locus associated with an argument toward the position associated with another argument. The SIGN-HUB tests were developed with the goal of assessing the comprehension of this phenomenon with a truth-value judgment task, in which the target sentence containing an agreeing verb appeared on the screen right after a non-linguistic clip describing a situation with at least two characters. Participants had to judge whether the target sentence matched the situation described in the clip or not (Aristodemo et al., 2020).

### Summary of Results: Long Distance vs. Sign Language Specific

In all the tests the results were clear: a delayed AoE had a lifelong impact on individuals’ language performance and/or competence.

As for the comprehension of *wh-*questions, only the results concerning LIS and LSF were analyzed so far. In LIS, native signers outperformed non-native not only in object questions, that were expected to be complex, but also in control questions, which were easy (Cecchetto et al., 2021). Even in this simple task, a difference emerged, confirming permanent effects of delayed exposure to sign language. For LSF, comparing language groups, a marginal difference was found between native and late learners, but a significant interaction emerged between this factor, the type of question and the subject/object condition. It was also found that the complexity provoked by object questions especially in which-questions particularly affected late learners of LSF. Importantly, in both LIS and LSF, early and late signers did not perform differently (Cecchetto et al., 2021; Hauser et al., in press).

The same consistent results have been found in the comprehension of role shift (RS) across the three languages: in LIS, native signers outperformed early and late signers both when the first-person pronoun appeared in subject and object position, with RS and without RS. In LSF, native signers outperformed early signers in all types of sentences in both conditions. Moreover, native signers outperformed late signers in all sentences with RS. This was not the case for sentences without RS, but one might speculate that this is because the late signers who performed worse in RS preferred by default the condition without RS. This might explain why late signers outperformed early signers in sentences without RS, and why the difference between late and native signers was not significant. In LSC, all groups had a good performance in sentences without RS. On the contrary, the performance in sentences with RS was more variable, but poor in native signers, and very poor in early and late learners. These results are attributed in the paper to a series of factors related to the non-manual markers associated to RS in the LSC tests, which were relatively subtle and might not have been clearly perceived by non-native signers (Aristodemo et al., in press).

The test on the comprehension of agreement with directional verbs, for which only LSF and LIS data were analyzed so far, also goes in the same direction: native signers outperformed non-native in LIS in the mismatch conditions. In LSF, instead, native signers were more accurate than non-native in both mismatch and match conditions. In general, no difference between early and late signers was found.

Finally, the test on relative clauses provides further evidence about the impact of AoE, and the special status of native signers. As for LSF, Hauser et al. (2021) report that for all three groups the difference between subject RCs (SRC) and object RCs (ORC) was significant, such that subject RCs were understood more easily. In the comprehension of ORCs, native signers performed significantly better than late learners and performed better than early learners in SRCs, but not significantly so. No significant difference was found between early and late learners. In LIS, native signers significantly outperformed early learners in SRCs, and they outperformed both early and late learners in ORCs. The difference between early and late learners was not significant.

As for LSC, the results obtained went even beyond expectations about AoE affecting adults’ performance, and raised interesting questions. Again, SRCs were significantly better understood than object RCs across all three groups. As for ORCs, the difference between late and early learners only approached significance, while there was no significant difference between native signers and early learners. Late learners had a significantly lower performance than native signers. Interestingly, non-native learners were *below chance* when it came to ORCs, suggesting that non-native signers interpreted ORCs as SRCs. As discussed in detail in Hauser et al. (2021), this seems to represent an extreme case of AoE effect, where the difference in AoE produces a difference in grammar, not just in performance, with native signers having both SRCs and ORCs in their grammar while non-native signers not allowing ORCs at all in LSC.

The results we just outlined can be summarized in Table 4. For each language, the first column in Table 4 indicates for every phenomenon investigated whether we found a significant difference in at least one condition of the tests between native and non-native signers. The second column summarizes for each phenomenon whether we found a significant difference in at least one condition of the tests between early and late learners.

**TABLE 4:** Summary of the SIGN-HUB tests where native signers significantly outperformed non-native and where early learners significantly outperformed late learners in at least one condition of the tests.

	Native vs. Non-native			Early vs. Late		
	LIS	LSC	LSF	LIS	LSC	LSF
*Wh-*question comprehension	✓	NA	×	×	×	×
Role shift comprehension	✓	✓	✓	×	×	×
Directional verb comprehension	✓	NA	✓	×	×	×
Relative clause comprehension	✓	✓	✓	×	×	×

Table 4 clearly indicates that language exposure from birth is an important factor in determining language competence in the syntactic phenomena that were investigated. It also points at the importance of *nativeness* over simple earliness of first language exposure. These results have been obtained in sign languages that differ significantly in the syntactic domains under investigation. Nevertheless they are fully comparable as far as the effect of *nativeness* and AoE is concerned, since they have been obtained with comparable populations of signers divided according to the same criteria in three groups: native signers, defined as signers exposed to sign language from birth and with at least one Deaf signing parent; early learners, defined as been exposed to sign language between the age of 1 and 5 (included); late learners, defined as been exposed to sign language between the age of 6 and 15 years.

It is important to underline, though, that the effect of *nativeness* is not found to the same extent in every condition and in every language tested. This is mainly due to the amount of population tested (less than 15 people for each AoE group in each language). Nevertheless, the effect is overall consistent.

## Discussion

Summarizing the main findings of the tests described in the preceding section, we can conclude that a delayed AoE has a direct impact on syntactic competences. This conclusion holds both for those linguistic phenomena that are widely known to be sensitive to language acquisition disruption, such as the comprehension of long-distance dependencies (assessed in the SIGN-HUB tests through the comprehension of relative clauses and content questions), and for grammatical features that are more specific to the signing modality, such as the comprehension of role shift and of agreement with directional verbs. Moreover, this conclusion holds true across different sign languages, notwithstanding important syntactic differences across constructions.

Remember that the question at stake in this paper is whether the traditional centrality that is assigned to native signers in the linguistic literature makes sense in relation to the signing populations, where native signers are a small minority, certainly not representative of the general population of signers.

### Native Signers Are Different

In all the phenomena that were investigated, a significant difference emerged between native and early learners. This pattern appears to strongly confirm that there is a categorial effect of being a native signer that goes beyond simple AoE, a more continuous measure. There are at least two possible interpretations for this finding, not necessarily mutually exclusive.

A first interpretation is that being a native signer goes beyond timing of exposure, determining the quality and quantity of the input: native signers are likely to be the only population which is exposed in a natural environment to an fully fledged input, which might be lacking in school environments, where non-native signers are usually exposed to sign language. It is thus likely that the better performance of native signers is related to this qualitative and quantitative difference in the input received.

While this is certainly true, it cannot be the whole story. First of all, keep in mind that even Deaf parents are not a uniform class, and many might have themselves been exposed to sign language at a late period in life, thus providing an input that is not qualitatively different, at least as far as pure linguistic properties are concerned, from the input the general population is exposed to (cf. Lillo-Martin, 2021, and Singleton and Newport, 2004). Second, this “qualitative/quantitative” explanation would not extend to other findings pointing at a privilege of those children who are very early exposed to language as opposed to early exposed ones, no matter the family environment they are immersed in.

Friedmann and Szterman (2006) studied the competence in Hebrew of a group of hearing-impaired Hebrew-speaking (hence orally trained) children, all growing in hearing families under very similar circumstances. They found that individual performance in comprehension of long-distance dependencies in Hebrew was strongly correlated with the age of intervention: only children who received hearing aids before the age of 18 months performed well in the comprehension tasks. No other factors, such as the degree of hearing loss or the type of hearing device, significantly affected their performance. These findings indicate that something critical happens between birth and 1,5 years of age for syntax: in other words, they suggest that the critical period for first language syntactic competence is very early.

Friedmann and Rusou (2015) discuss the important issue of the effect of AoE in syntactic competences in a review paper, where they underline that most of the studies of a critical period for language acquisition test the acquisition of a second language, when one language has already been acquired. They suggest that a critical period for acquiring a first language is crucially different and earlier in time, and that for the acquisition of syntax it is the first year of life. While these results were only available until now with respect to spoken language inputs, the SIGN-HUB tests’ contribution confirms the existence of this critical threshold also for sign language, which is not surprising considering that sign languages are natural languages just as any other, governed by the same bioprogram.

Be that as it may, this conclusion has important practical consequences that should be underlined in the most explicit way. Whether hearing aided or not, in order to guarantee unhindered language acquisition, deaf children should be exposed to sign language as early as possible, ideally from birth.

### But Maybe Not Too Different

A question that we have not yet discussed is whether the lower performance that was captured in non-native signers is due to a competence gap (non-native signers have developed a different grammar) or to a performance gap (the resources necessary for computation are scarcer in non-native signers but the internal grammar is the same). Take the Subject Advantage in long-distance dependencies. The SIGN-HUB data show that this effect is stronger in non-native than in native signers. In the acquisition literature, the fact that the Subject Advantage in relative clauses and *wh*-questions gets reduced with age in simple picture matching tasks has been interpreted in terms of lower computational resources in young children. A similar explanation might be adopted here. Comprehending a first language acquired with a delay involves a bigger effort and this emerges in complex tasks. It was also noticed in Aristodemo et al. (in press) that a co-factor determining the particularly low performance of LSC non-native signers in the role shift comprehension task is the fact that in LSC stimuli non-manual markers were relatively subtle and might have not been noticed by non-native signers. This as well goes in the direction of a performance account.

If this were all that was found, we could conclude that native signers are different in that their performance is not affected by scarcity of resources, and they are more reliable as a source of linguistic information because their performance more directly reflects their grammatical competence. However, if we take a closer look at LSC for the relative clauses task, the picture appears to be different. In this language it was found that the Subject Advantage is so strong as to take the shape of a categorical difference between the grammar of native signers and that of non-native signers, who systematically misunderstand ORCs. The overall results suggest that while native signers have both SRCs and ORCs in their grammar, non-native signers do not allow ORCs at all in LSC. The fact that different varieties of languages realize different steps of the Accessibility Hierarchy of Keenan and Comrie (1977), which states that subject positions are more accessible than object positions in relativization, should not come as a surprise given the exceptional circumstances of access to language experienced by a large part of the deaf population. In fact, this finding, which replicates language internally the conclusion based on the typological literature, appears as an extreme case of AoE effect (Hauser et al., 2021).

If this is true, however, the question of the reliability of native signers gets partially reversed: if they sign a qualitatively different language, that is indeed a tight minority language within the community of signers, how can we capitalize on their language for description, pedagogical tools, standardization procedures, or language assessment? As for the latter, these findings advocate for the development of specific baselines at least distinguishing native from non-native signers. As for language description and its practical uses, the findings of the SIGN-HUB tests suggest that the common practice of relying exclusively on native signers should be complemented with a careful consideration of possible variations in different populations, crucially related to AoE.

## Conclusion

In this paper we provide an overview of the morpho-syntactic SIGN-HUB assessment tests, a large-scale cross-linguistic study investigating comprehension across different sign languages and different syntactic phenomena to shed light on the notion of native signer. By relying on the same criteria to define native, early and late signers, the SIGN-HUB tests were able to provide new evidence that being exposed to sign language from birth has a permanent effect on language competence. In the syntactic tasks that were administered, native signers significantly outperformed non-native signers in a consistent way in most of the conditions tested.

While these results confirm that native signers perform differently from non-native signers, early learners included, they also suggest that at least for some phenomena and for some languages (and in particular for relative clauses in LSC) non-native learners develop a grammar that is significantly and qualitatively different from that of native signers. Overall, these results reaffirm the importance of native signers within the signing community, but also challenge the generalized use of the notion of native speaker/signer as the baseline for language description and language assessment. This is a crucial point when assessing clinical populations and should be considered through the life span, given that a delay in language exposure during childhood has permanent effects also in adulthood.

## Data Availability Statement

The original contributions presented in the study are publicly available. This data can be found here: for Hauser et al. (in press) https://osf.io/paj9n/?view_only=c9eaff3ba5a541cf9829a7de59a82e56; for Cecchetto et al. (2021) https://osf.io/g5cm9/; for Aristodemo et al. (in press) https://osf.io/emp6g/; for Hauser et al. (2021) https://osf.io/5bdu2/.

## Ethics Statement

Ethical review and approval, and written informed consent, were not required for the current study in accordance with the local legislation and institutional requirements. As for the SIGN-HUB tests discussed in this article, they were reviewed and approved by for France (CERES, IRB n°. 20163400001072), for Italy (Milan Bicocca Ethical Committee, prot. n°. 0019845/16), for Spain (Parc de Salut MAR – Clinical Research Ethics Committee, prot. n°. 2016/6715/I). Participants provided their written informed consent to participate in these studies. Please refer to each specific paper for further details.

## Author Contributions

All authors contributed to the conceptualization of the manuscript. GZ wrote the first draft of the manuscript with the help of CD. GZ revised the manuscript with the help of CD and BG. All authors approved the final manuscript. CD, CC, and JQ supervised the project and acquired the funding.

## Conflict of Interest

The authors declare that the research was conducted in the absence of any commercial or financial relationships that could be construed as a potential conflict of interest.

## Publisher’s Note

All claims expressed in this article are solely those of the authors and do not necessarily represent those of their affiliated organizations, or those of the publisher, the editors and the reviewers. Any product that may be evaluated in this article, or claim that may be made by its manufacturer, is not guaranteed or endorsed by the publisher.

## Footnotes

1 Following standard practice, in this paper we use the word “deaf” with low case “d” to refer to the audiological condition of a deaf individual. The word “Deaf” with capital “D”, instead, will be used to refer to the deaf members of a community who use a sign language, with its own cultural identity.

2 Costello et al. (2008) used data provided by the Spanish National Association of the Deaf and the Spanish Statistical Institute.

3 Concerning the onset of deafness, participants self-reported that it was never later than 3 years old (LIS: *M* = 3.5 months, LSC: *M* = 5.6 months, LSF: *M* = 3.7 months).

4 Questionnaires were written, but a signing person was present so participants who had doubts could ask for a translation.

5 A secondary goal was to start developing clinical tests to assess language impairment in Deaf adults. With this purpose in mind, two lexical comprehension tasks were also designed in order to detect potential impairments at the lexical level, in the phonological system and in the semantic one, respectively. The question of the role of AoE was marginal in these tasks, since the literature suggests that AoE does not have an impact on the size of the lexicon (i.e., on accuracy in lexical comprehension tasks, e.g., Carreiras et al., 2008; Dye and Shih, 2009).

6 All SIGN-HUB tests are available under request. For more information, see https://www.sign-hub.eu/assessment/welcome-page-assessment

7 Three characters pictures were used in the tests on relative clauses (Giustolisi et al., 2019, for LIS, Zorzi et al., 2019a, for LSC and Hauser et al., 2019, for LSF) and wh-interrogatives (Checchetto et al., 2019, for LIS, Zorzi et al., 2019b, for LSC and Aristodemo et al., 2019, for LSF).
